# Lymphangiogenesis in hypercholesterolemic skin: A comparative study of xanthomatous and non-xanthomatous lesions

**DOI:** 10.1016/j.xjidi.2026.100475

**Published:** 2026-04-08

**Authors:** Enkhtuul Gantumur, Nao Itai, Kazuki Takagaki, Nobuyuki Mitsukawa, Shinsuke Akita, Kentaro Kajiya

**Affiliations:** 1 MIRAI Technology Institute, Shiseido Co., Ltd., Yokohama, Japan; 2 Department of Plastic, Reconstructive, and Aesthetic Surgery, Chiba University, Japan

**Keywords:** Dermal lymphatics, Hypercholesterolemia, LDL cholesterol, Lymphangiogenesis, Xanthelasma palpebrarum

## Abstract

While hypercholesterolemia is the primary driver of atherosclerosis, emerging evidence indicates that lymphatic vessels are critical for cholesterol transport and become impaired under hyperlipidemic conditions. Xanthelasma palpebrarum, characterized by cholesterol-laden yellowish plaques on the eyelids, is a cutaneous manifestation of dyslipidemia, yet the relationship between local cholesterol accumulation and dermal lymphatic function remains elusive. Here, we compare the lymphatic vessel morphology of xanthomatous and non-xanthomatous skin lesions in patients with various levels of serum low-density lipoprotein cholesterol. Dermal lymphatic vessel abundance was positively correlated with serum low-density lipoprotein cholesterol levels in both lesion types. Elevated low-density lipoprotein cholesterol was associated with upregulated VEGF-C expression in macrophages and increased lymphatic proliferation, indicating aberrant lymphangiogenesis in hypercholesterolemic skin. These findings reveal a previously unrecognized link between systemic cholesterol metabolism and lymphatic remodeling in human skin. The observed lymphangiogenic response may represent a compensatory mechanism for impaired cholesterol clearance or may contribute to the pathogenesis of xanthoma.

## Introduction

Hypercholesterolemia, characterized by elevated serum low-density lipoprotein cholesterol (LDL-C) levels, is a major modifiable risk factor for cardiovascular disease (Collado et al, 2021). Beyond the well-established role of elevated LDL-C in atherosclerosis, emerging evidence indicates that the systemic effects extend beyond the vascular system to significantly impact cutaneous tissues, leading to the formation of xanthomas consisting of accumulations of lipid-laden macrophages, known as foam cells (Wu et al, 2014). The most clinically apparent dermatological manifestation is xanthelasma palpebrarum—distinctive xanthomatous lesions that occur around the eyelids (Aljenedil et al, 2018; Li et al, 2018; Zak et al, 2014). Current models of xanthoma pathogenesis, however, fail to account for either the preferential localization of lesions to specific anatomical sites, particularly the eyelids, or the considerable variability in lesion development among individuals with similar cholesterol profiles. Furthermore, xanthelasma palpebrarum frequently persists despite aggressive lipid-lowering therapy, suggesting that systemic cholesterol reduction alone may be insufficient for lesion resolution (Christoffersen et al, 2011; Rohrich et al, 2002).

The lymphatic system plays a critical role in cholesterol homeostasis by mediating reverse cholesterol transport, facilitating the removal of excessive cholesterol from peripheral tissues to the liver (Martel et al, 2013; Randolph and Miller, 2014). Previous studies using mouse models have demonstrated that severe hypercholesterolemia impairs lymphatic vessel function, with apolipoprotein E (ApoE)-deficient mouse showing lymphatic vessel dilation, decreased smooth muscle cell coverage, and functional impairment (Lim et al, 2009). Additionally, in vitro studies have revealed that oxidized LDL-C directly suppresses lymphangiogenesis in cultured lymphatic endothelial cells via CD36 signaling, while native LDL-C stimulates tube formation (Singla et al, 2021). However, whether these direct effects on lymphatic vessels observed in mouse models or isolated culture systems translate to human tissue, where complex cellular interactions occur, remain elusive. Moreover, the effects of moderate, naturally occurring elevated LDL-C on lymphatic vessels in human skin have not been systematically investigated. Eyelid skin, where xanthomas preferentially develop, represents a particularly relevant site to examine these relationships, as its thin dermis and dense vascular networks may render it especially vulnerable to metabolic perturbations and lymphatic alterations (Kajiya et al, 2018).

To systematically investigate the relationship between hypercholesterolemia and structural as well as functional alterations in cutaneous lymphatic vessels, we conducted a comprehensive analysis of xanthomatous and non-xanthomatous eyelid skin from individuals with various levels of serum LDL-C. Our findings indicate that elevated serum LDL-C induces a cascade of pathological changes in skin tissue, including epidermal hyperplasia, inflammatory cell infiltration, and aberrant lymphangiogenesis, with xanthomas representing the most advanced stage of this continuum rather than discrete pathological entities. Understanding these relationships is crucial for developing a comprehensive picture of how elevated cholesterol affects peripheral tissues and the pathogenic mechanisms underlying xanthoma formation.

## Results

### Study population and baseline characteristics

**Figure 1:**
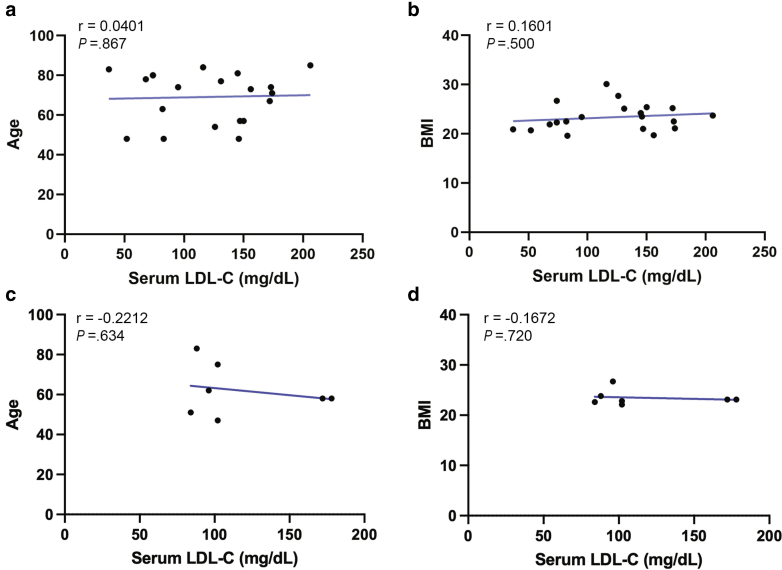
**No significant correlation was observed between age or BMI and serum LDL-C levels in non-xanthomatous and xanthomatous skin.** The relationship between **(a, c)** age or **(b, d)** BMI and serum LDL-C levels in **(a, b)** non-xanthomatous (n = 20) and **(c, d)** xanthomatous skin (n = 7). Student’s *t*-test for the Pearson correlation coefficient was utilized. *P* < .05 was considered statistically significant. BMI, body mass index; LDL-C, low-density lipoprotein cholesterol.

To investigate serum LDL-C level-related changes in hypercholesterolemic skin, we conducted a cross-sectional study examining both xanthomatous and non-xanthomatous skin lesions. Skin samples were obtained from subjects based on comprehensive blood biochemical profiles, medication history, and dermatological examination findings. The study cohort consisted of 20 healthy adults with non-xanthomatous skin (mean age, 69 ± 13 years; serum LDL-C range, 120 ± 47 mg/dL) and 7 patients with xanthomatous skin (mean age, 62 ± 13 years; serum LDL-C range, 117 ± 40 mg/dL) (Table 1). Based on the Japan Atherosclerosis Society guidelines for prevention of atherosclerotic cardiovascular diseases 2022 (Okamura et al, 2024), samples were categorized as low LDL-C (< 120 mg/dL) or high LDL-C (≥ 120 mg/dL) for comparative analyses throughout this study. Subjects taking cholesterol-lowering medication were excluded from the analysis of non-xanthomatous skin. No significant correlations were observed between serum LDL-C levels and either age or body mass index, suggesting no confounding of the relationship between LDL-C levels and skin changes by these variables (Figure 1a–d).

**Table 1:** Demographic Characteristics of the Skin Samples

Lesion Type		Non-Xanthomatous (n = 20)	Xanthomatous (n = 7)
Parameter	Unit	Mean ± SD	Mean ± SD
Age	Y	69.1 ± 12.9	62.0 ± 12.8
Sex	F/M	10/10	5/2
BMI	kg/m^2^	23.4 ± 2.7	23.4 ± 1.5
LDL-C	mg/dL	120.4 ± 47.1	117.4 ± 39.9
HDL-C	mg/dL	58.7 ± 19.3	65.1 ± 9.65
TG	mg/dL	105.4 ± 74.0	101.8 ± 45.7

Abbreviations: BMI, body mass index; HDL-C, high-density lipoprotein cholesterol; LDL-C, low-density lipoprotein cholesterol; TG, triglycerides.

### Characterization of non-xanthomatous and xanthomatous skin

**Figure 2:**
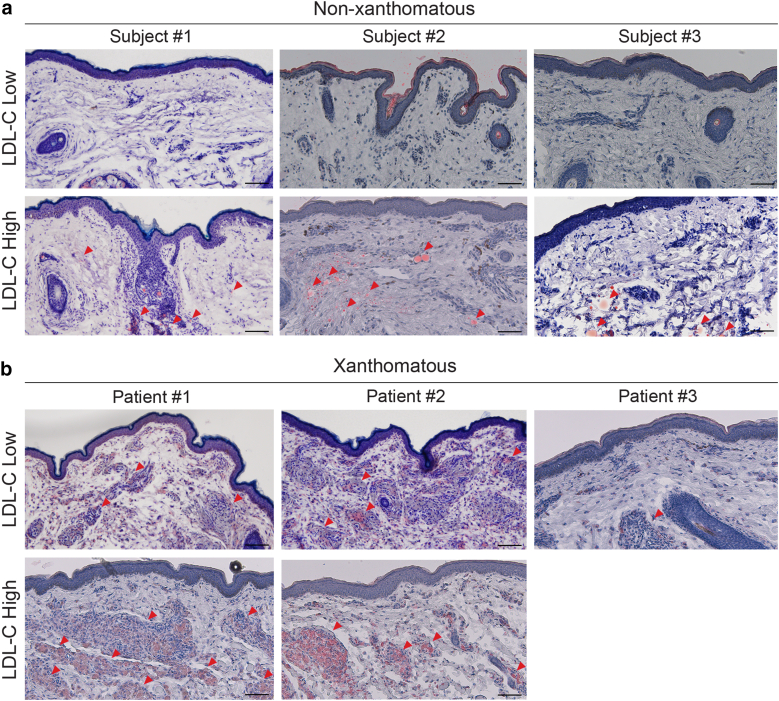
**Lipid accumulation is increased in non-xanthomatous and xanthomatous skin with elevated serum LDL-C levels.** Representative Oil Red O staining images of **(a)** non-xanthomatous and **(b)** xanthomatous skin samples with low or high serum LDL-C levels. Non-xanthomatous skin samples were obtained from subjects with low LDL-C (68 mg/dL, 82 mg/dL, 112 mg/dL < 120 mg/dl) and high LDL-C (145 mg/dL, 147 mg/dL, 172 mg/dL, ≥ 120 mg/dL) levels. Xanthomatous skin samples were obtained from patients with low LDL-C (84 mg/dL, 96 mg/dL, 102 mg/dL, < 120 mg/dL) and high LDL-C (172 mg/dL, 178 mg/dL, ≥ 120 mg/dL) levels. Scale bars = 100 μm. Red arrows indicate lipid deposition in panel **(a)** and lipid-rich foam cells in panel **(b)**. LDL-C, low-density lipoprotein cholesterol.

The lipid content of non-xanthomatous and xanthomatous skin was assessed by means of Oil Red O staining. Non-xanthomatous skin of subjects with elevated serum LDL-C levels exhibited relatively modest lipid deposition, while no lipid droplets were detected in the case of subjects with low serum LDL-C (Figure 2a). Consistent with previous findings (Govorkova et al, 2018), lipid-rich foam cell accumulation was present in xanthomatous skin (Figure 2b). Notably, specimens from patients with elevated serum LDL-C showed significantly greater deposition. These observations suggest that high serum LDL-C plays a role in the pathogenesis of skin xanthoma, which may represent a late stage of initially subclinical lipid-driven cutaneous remodeling in response to systemic hypercholesterolemia.

### Serum LDL-C levels are positively correlated with epidermal changes in non-xanthomatous skin

**Figure 3:**
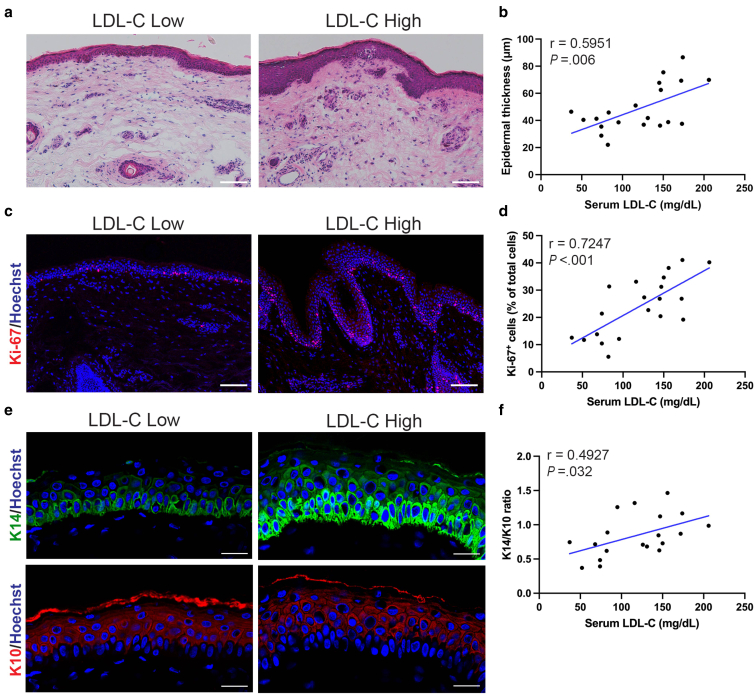
**Elevated serum LDL-C levels are associated with epidermal hyperplasia in non-xanthomatous skin.** (**a**) Representative histological images of skin samples from subjects with low LDL-C (52 mg/dL, < 120 mg/dL) and high LDL-C (206 mg/dL, ≥ 120 mg/dL) levels. Bars = 100 μm. **(b)** Correlation between the epidermal thickness and serum LDL-C levels (n = 20; Pearson correlation, *r* = 0.5951, *P* = .006) **(c)** Representative immunofluorescence staining of Ki-67 (red) in skin samples from subjects with low LDL-C (52 mg/dL, < 120 mg/dL) and high LDL-C (172 mg/dL, ≥ 120 mg/dL) levels. Scale bars = 100 μm. **(d)** Correlation between the percentage of Ki-67^+^ cells relative to the total epidermal cells and serum LDL-C levels. (n = 20; Pearson correlation, *r* = 0.7247, *P* < .001) **(e)** Representative immunofluorescence staining of K14 (green) and K10 (red) in skin samples from subjects with low LDL-C (74 mg/dL, < 120 mg/dL) and high LDL-C (172 mg/dL, ≥ 120 mg/dL) levels. Scale bars = 25 μm. (**f**) Correlation between the K14/10 ratio in epidermis and serum LDL-C levels (n = 19; Pearson correlation, *r* = 0.4927, *P* = .032). Nuclei were stained with Hoechst (blue). Student’s *t*-test for the Pearson correlation coefficient was utilized. *P* < .05 was considered statistically significant. K10, keratin 10; K14, keratin 14; LDL-C, Low-density lipoprotein cholesterol.

To assess the early skin changes in response to systemic hypercholesterolemia, we performed histological and immunohistochemical analysis of non-xanthomatous skin samples from normal individuals with various serum LDL-C levels. H&E staining revealed a marked increase in epidermal thickness in individuals with high serum LDL-C levels, and quantitative analysis confirmed a significant positive correlation (*r* = 0.5951, *P* = .006) (Figure 3a and b). To further assess whether these changes are a result of hyperproliferation, we performed immunofluorescence staining for Ki-67. The percentage of Ki-67^+^ cells relative to the total epidermal cells was significantly elevated in skin samples from individuals with higher serum LDL-C levels, indicating increased proliferative activity (*r* = 0.7247, *P* < .001) (Figure 3c and d). To characterize the epidermal differentiation status, we examined the ratio of keratin 14 (K14), as a basal cell marker, to keratin 10 (K10), as a differentiated cell marker (Campbell et al, 2025; Ota et al, 2014). The K14/K10 ratio was significantly correlated with serum LDL-C levels (*r* = 0.4927, *P* = .032), indicating hyperproliferative epidermis (Figure 3e and f).

**Figure 4:**
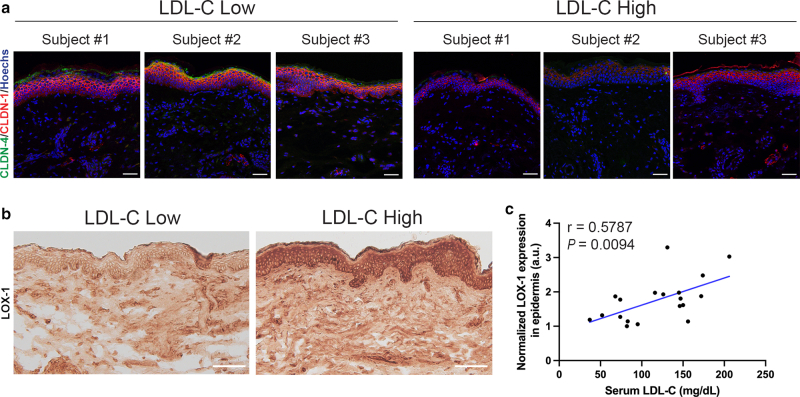
**Altered skin barrier integrity and LOX-1 expression in relation to serum LDL-C levels in non-xanthomatous skin.** (**a**) Representative immunofluorescence staining of CLDN-1 (red) and CLDN-4 (green) in non-xanthomatous skin samples from subjects with low LDL-C (68 mg/dL, 74 mg/dL, 74 mg/dL, < 120 mg/dL) and high LDL-C (150 mg/dL, 156 mg/dL, 206 mg/dL, ≥ 120 mg/dL) levels. Scale bars = 25 μm. **(b)** Representative immunohistochemical staining of LOX-1 in skin samples from subjects with low LDL-C (74 mg/dL, < 120 mg/dL) and high LDL-C (145 mg/dL, ≥ 120 mg/dL) levels. Scale bars = 100 μm. **(c)** Correlation between the expression of LOX-1 in epidermis and serum LDL-C levels (n = 19; Pearson correlation, *r* = 0.5787, *P* =.009). Claudin expression levels showed no significant correlation with serum LDL-C levels (data not shown). Nuclei were stained with Hoechst (blue). Student’s *t*-test for the Pearson correlation coefficient was utilized. *P* < .05 was considered statistically significant. CLDN, claudin; LDL-C, Low-density lipoprotein cholesterol; LOX-1, lectin-like oxidized LDL receptor-1.

Given that epidermal hyperproliferation and altered differentiation can compromise barrier integrity, we next assessed the expression of claudin-1 (CLDN-1) and claudin-4 (CLDN-4), tight junction proteins critical for epidermal barrier function (Furuse et al, 2002). Representative immunofluorescence images from subjects with varying serum LDL-C revealed qualitatively reduced expression of both CLDN-1 and CLDN-4 in skin with elevated LDL-C levels, suggesting that barrier function might be compromised under conditions of hypercholesterolemia (Figure 4a).

To further explore the molecular changes associated with cholesterol accumulation in hypercholesterolemic skin, we examined the expression of lectin-like oxidized LDL receptor-1 (LOX-1), a key scavenger receptor involved in cholesterol uptake (Yoshida et al, 1998). Immunohistochemical staining showed a marked increase in LOX-1 expression in the epidermis of individuals with high LDL-C levels, and quantitative analysis revealed a significant positive correlation (*r* = 0.5787, *P* = .009) (Figure 4b and c). Taken together, these results indicate that systemic hypercholesterolemia is associated with altered epidermal homeostasis, characterized by increased proliferation, disrupted differentiation, enhanced cholesterol uptake receptor expression, and potential impairment of barrier function.

### Serum LDL-C levels are positively correlated with M1 macrophage accumulation in non-xanthomatous skin

**Figure 5:**
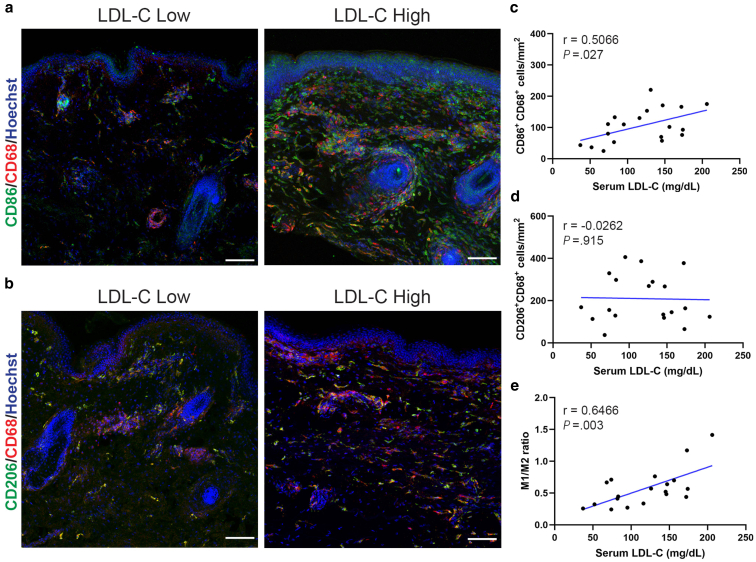
**Serum LDL-C levels are positively correlated with the number of M1 macrophages in non-xanthomatous skin.** Representative immunofluorescence staining of **(a)** CD86 (red) and CD68 (green); **(b)** CD206 (red) and CD68 (green) in skin samples from subjects with low LDL-C (82 mg/dL, < 120 mg/dL) and high LDL-C (147 mg/dL, ≥ 120 mg/dL) levels. Scale bars = 100 μm. **(c)** Correlation between the number of CD86^+^ CD68^+^ M1 macrophages in the dermis and serum LDL-C levels (n = 19; Pearson correlation, *r* = 0.5066, *P* = .027). **(d)** Correlation between the number of CD206^+^ CD68^+^ M2 macrophages in the dermis and serum LDL-C levels (n = 19; Pearson correlation, *r* = -0.0262, *P* = .915). **(e)** Correlation between the M1/M2 ratio and serum LDL-C levels (n = 19; Pearson correlation, *r* = 0.6466, *P* = .003). Nuclei were stained with Hoechst (blue). Student’s *t*-test for the Pearson correlation coefficient was utilized. *P* < .05 was considered statistically significant. LDL-C, Low-density lipoprotein cholesterol.

The previous observations suggested active cholesterol uptake and potential lipid accumulation in hypercholesterolemic skin. Since macrophages play a critical role in lipid clearance and can adopt distinct phenotypes in response to lipid loading, we next assessed macrophage polarization in the dermis using immunofluorescence co-staining for CD68 (pan-macrophage marker) with CD86 (M1 pro-inflammatory marker) or CD206 (M2 anti-inflammatory marker) (Spencer et al, 2010). In high LDL-C skin samples, there was a marked increase in CD86^+^CD68^+^ M1 macrophages compared to the low LDL-C skin samples, while CD206^+^CD68^+^ M2 macrophages showed no apparent difference (Figure 5a and b). Quantitative analysis confirmed a significant positive correlation between serum LDL-C levels and the number of dermal M1 macrophages (CD86^+^CD68^+^) (*r* = 0.5066, *P* = .027; Figure 5c). In contrast, the number of M2 macrophages (CD206^+^CD68^+^) showed no significant correlation with LDL-C levels (Figure 5d). Consequently, the M1/M2 macrophage ratio was significantly elevated in individuals with higher LDL-C levels (*r* = 0.6466, *P* = .003; Figure 5e). These findings reveal that hypercholesterolemia induces subclinical dermal inflammation in apparently normal skin, characterized by preferential M1 macrophage polarization that may precede visible xanthoma formation.

### Serum LDL-C levels are positively correlated with alteration of dermal lymphatics in non-xanthomatous skin

**Figure 6:**
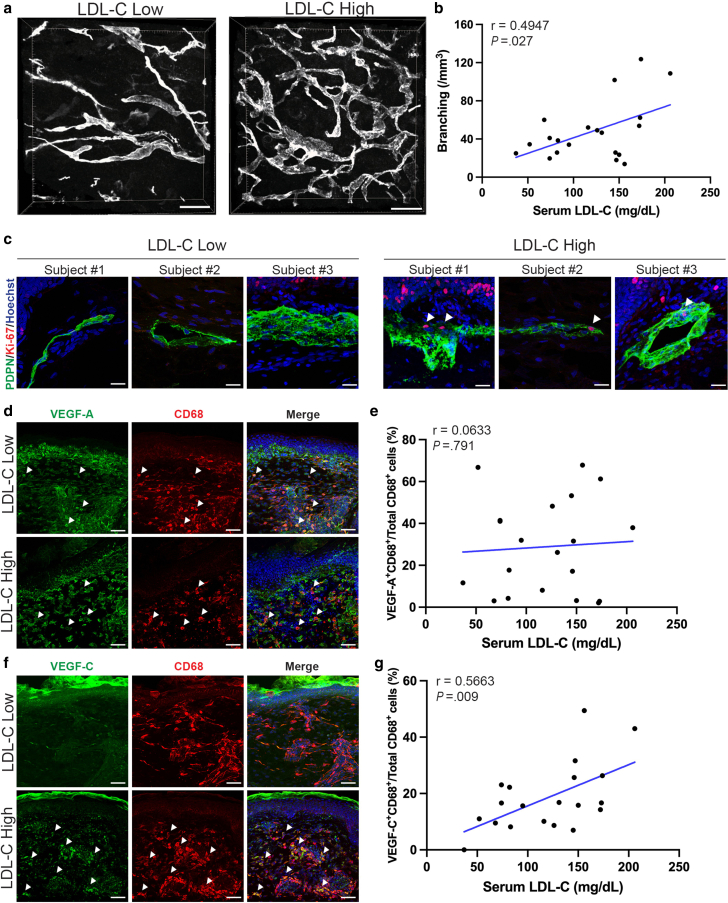
**Phenotype of dermal lymphatics alters with serum LDL-C levels in non-xanthomatous skin.** (**a**) Representative 3D images of PDPN^+^ lymphatic capillaries in non-xanthomatous skin samples from subjects with low LDL-C (82 mg/dL, < 120 mg/dL) and high LDL-C (174 mg/dL, ≥ 120 mg/dL) levels. Scale bars = 200 μm. **(b)** Correlation between the branching of lymphatic capillaries and serum LDL-C levels (n = 20; Pearson correlation, *r* = 0.4947, *P* = .027). **(c)** Ki-67^+^ (red) PDPN^+^ (green) dermal lymphatics were identified in skin samples from subjects with high LDL-C (145 mg/dL, 172 mg/dL, 174 mg/dL, ≥ 120 mg/dL) serum LDL-C levels, indicating the proliferation of lymphatic capillaries (arrows). Scale bars = 25 μm. **(d)** Representative immunofluorescence staining of VEGF-A (green) and CD68 (red) in skin samples from subjects with low LDL-C (74 mg/dL, < 120 mg/dL) and high LDL-C (172 mg/dL, ≥ 120 mg/dL) levels. Arrows indicate double-positive macrophages. Scale bars = 50 μm. **(e)** Correlation between the percentage of VEGF-A^+^ CD68^+^ macrophages (relative to total CD68^+^ macrophages) and serum LDL-C levels (n = 20; Pearson correlation, *r* = 0.0633, *P* = .791). **(f)** Representative immunofluorescence staining of VEGF-C (green) and CD68 (red) in skin samples from subjects with low LDL-C (83 mg/dL, < 120 mg/dL) and high LDL-C (206 mg/dL, ≥ 120 mg/dL) levels. Arrows indicate double-positive macrophages. Scale bars = 50 μm. **(g)** Correlation between the percentage of VEGF-C^+^ CD68^+^ macrophages (relative to total CD68^+^ macrophages) and serum LDL-C levels (n = 20; Pearson correlation, *r* = 0.5663, *P* = .009). Nuclei were stained with Hoechst (blue). Student’s *t*-test for the Pearson correlation coefficient was utilized. *P* < .05 was considered statistically significant. LDL-C, Low-density lipoprotein cholesterol; PDPN, podoplanin.

Given that macrophage-mediated inflammation can trigger tissue remodeling and that lymphatic vessels play an important role in cholesterol clearance, we investigated structural changes in dermal lymphatic capillaries. Whole-mount immunofluorescence for podoplanin (PDPN), a marker of lymphatics (Breiteneder-Geleff et al, 1999), was performed. Skin samples from individuals with elevated serum LDL-C exhibited more complex and extensively branched dermal lymphatic networks compared to samples from low LDL-C individuals (Figure 6a). Morphometric analysis confirmed a positive correlation between serum LDL-C levels and lymphatic capillary branching (*r* = 0.4947, *P* = .027) (Figure 6b).

To understand the mechanism of increased branching in hypercholesterolemic skin, double immunofluorescence staining of Ki-67 and PDPN was performed. Proliferative lymphatic endothelial cells (Ki-67^+^PDPN^+^) were readily identified in high LDL-C skin samples, indicating the involvement of prominent lymphangiogenesis (Figure 6c). Given the established role of macrophage-derived VEGF-A and VEGF-C in lymphatic vessel function (Ji, 2012; Maruyama et al, 2005), we next examined their expression in dermal macrophages. Immunofluorescence co-staining for VEGF-A and CD68 revealed the presence of double-positive macrophages in both low and high LDL-C skin samples, with no significant correlation between serum LDL-C (*r* = 0.0633, *P* = .791), suggesting that VEGF-A expression is independent of cholesterol status (Figure 6d and e). In contrast, co-staining for VEGF-C and CD68 demonstrated a marked increase in VEGF-C–positive macrophages specifically in elevated LDL-C skin samples, with a significant positive correlation (*r* = 0.5663, *P* = .009) (Figure 6f and g). These data indicate that elevated LDL-C specifically upregulates macrophage-derived VEGF-C, suggesting a VEGF-C-dependent remodeling of the dermal lymphatic network in non-xanthomatous skin.

### Xanthomatous lesions exhibit lymphatic remodeling similar to that in non-xanthomatous hypercholesterolemic skin

**Figure 7:**
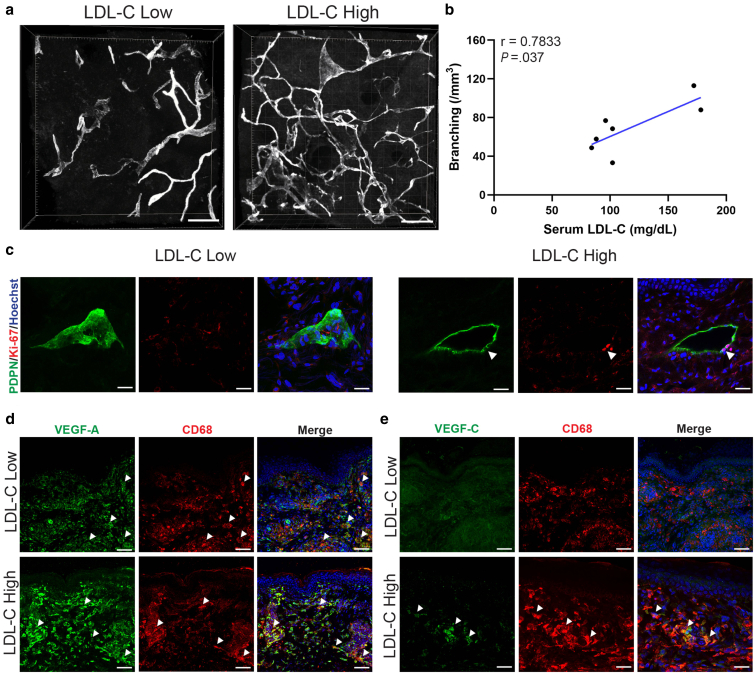
**Phenotypes of dermal lymphatics alter with serum LDL-C levels in xanthomatous skin.** (**a**) Representative 3D images of PDPN^+^ lymphatic capillaries in xanthomatous skin samples from patients with low LDL-C (96 mg/dL, < 120 mg/dL) and high LDL-C (172 mg/dL, ≥ 120 mg/dL) levels. Scale bars = 200 μm. **(b)** Correlation between the branching of lymphatic capillaries and serum LDL-C levels in xanthomatous skin samples (n = 7; Pearson correlation, *r* = 0.7833, *P* = .037). **(c)** Ki-67^+^ (red) PDPN^+^ (green) dermal lymphatics were identified in xanthomatous skin from a patient with a high LDL-C level (172 mg/dL, ≥ 120 mg/dL), indicating the proliferation of lymphatic capillaries (arrows). Scale bars = 25 μm. **(d)** Representative immunofluorescence staining of VEGF-A (green) and CD68 (red) in xanthomatous skin samples from patients with low LDL-C (96 mg/dL, < 120 mg/dL) and high LDL-C (172 mg/dL, ≥ 120 mg/dL) levels. Scale bars = 50 μm **(e)** Representative immunofluorescence staining of VEGF-C (green) and CD68 (red) in xanthomatous skin samples from patients with low LDL-C (96 mg/dL, < 120 mg/dL) and high LDL-C (172 mg/dL, ≥ 120 mg/dL) levels. Scale bars = 25 μm. Arrows in panels **(d)** and **(e)** indicate double-positive macrophages. Scale bars = 50 μm. Nuclei were stained with Hoechst (blue). Student’s *t*-test for the Pearson correlation coefficient was utilized. *P* < .05 was considered statistically significant. LDL-C, Low-density lipoprotein cholesterol; PDPN, podoplanin.

To determine whether the changes observed in non-xanthomatous hypercholesterolemic skin also occur in xanthomatous lesions, we analyzed xanthelasma palpebrarum skin samples from patients with various serum LDL-C levels. As found in non-xanthomatous skin, xanthomatous lesions from patients with high serum LDL-C levels exhibited increased lymphatic capillary branching that was positively correlated with serum LDL-C (*r* = 0.7833, *P* = .037) (Figure 7a and b). Xanthomatous lesions also contained Ki-67^+^PDPN^+^ proliferating lymphatic cells, suggesting active lymphangiogenesis (Figure 7c). Furthermore, co-localization of VEGF-A or VEGF-C with CD68^+^ macrophages in the dermis of high LDL-C xanthomatous lesions supported a role of macrophage-derived lymphangiogenic signaling in lesion development, paralleling changes seen in non-xanthomatous hypercholesterolemic skin (Figure 7d and e). These findings demonstrate that xanthomatous lesions exhibit similar lymphatic remodeling patterns to non-xanthomatous hypercholesterolemic skin, suggesting a common pathogenic mechanism driven by elevated cholesterol levels and macrophage-mediated lymphangiogenesis.

## Discussion

Extensive cholesterol accumulation within skin tissues under hypercholesterolemic conditions has been well-documented in various diet-fed mouse genotypes, such as ApoE knockout (Feingold et al, 1995; van Ree et al, 1995) and LDLr^-^^/^^-^, ApoA-I^-^^/^^-^ double knockout (Zabalawi et al, 2007). In these models, the skin emerged as the predominant site of cholesterol accumulation compared to other major organs, suggesting a particular vulnerability of cutaneous tissue to lipid deposition. Consistent with these findings, our investigation revealed increased lipid accumulation in both non-xanthomatous and xanthomatous skin from subjects with elevated serum LDL-C. However, a marked difference in lipid accumulation was observed between these groups despite similar serum LDL-C levels at the time of measurement. A possible explanation is that patients with xanthelasma palpebrarum had been receiving cholesterol-lowering medication, which likely reduced their circulating LDL-C levels at the time of measurement, though the duration and severity of prior hypercholesterolemic exposure might have already contributed to their xanthoma development (Wang et al, 2018). These observations suggest that systemic hypercholesterolemia initially triggers abnormal lipid accumulation in human skin through enhanced LDL uptake and impaired cholesterol efflux, with xanthoma formation representing a more advanced stage of this pathological process.

Beyond xanthoma formation, chronic systemic hypercholesterolemia has been associated with psoriasis, a chronic inflammatory skin disease (Luo et al, 2023; Wu et al, 2014). Imiquimod-induced ApoE-deficient mice show psoriasis-like skin phenotypes characterized by epidermal hyperplasia and upregulated LOX-1 expression in the epidermis (Shih et al, 2018; Xie et al, 2017). Furthermore, young adult ApoE-deficient mice show reduced skin barrier function due to their altered lipid composition (Martins Cardoso et al, 2019). We obtained similar findings and identified significant correlations with serum LDL-C levels. Thus, it is plausible that prolonged exposure to elevated LDL-C creates a systemic pro-inflammatory environment that not only promotes xanthoma development but also contributes to a broader spectrum of inflammatory conditions in the skin.

The positive correlation between the number of M1 macrophages in the dermis and serum LDL-C levels suggests a potential relationship between cholesterol levels and inflammatory processes in progressive skin inflammation. This finding is consistent with recent studies demonstrating that foamy macrophages in eruptive and tendon xanthomas exhibit a pro-inflammatory phenotype (Artieda et al, 2005; Huang et al, 2024). The mechanistic basis for this relationship is well-documented in atherosclerosis, where modified LDL-C, specifically oxidized LDL-C, serves as a potent stimulus for macrophage polarization toward M1 phenotype (Mushenkova et al, 2021; Seo et al, 2015; Wu et al, 2023). M1 macrophages create a local inflammatory microenvironment through robust production of pro-inflammatory cytokines, including TNF-α, IL-1β, and IL-6 (Artieda et al, 2005; Lara-Guzmán et al, 2018). Based on our findings and previous studies, we suggest that elevated serum LDL-C promotes dermal cholesterol accumulation, potentially leading to lipid oxidation and macrophage polarization. This inflammatory cascade may perpetuate chronic inflammation and contribute to the progression from subclinical lipid accumulation to clinically apparent xanthomas.

While numerous studies have examined the relationship between cholesterol homeostasis and lymphatic function in mouse models (Davis et al, 2023; Huang et al, 2015; Lim et al, 2013; Martel et al, 2013), a direct link between serum cholesterol and lymphatics in human skin has not been previously reported. To our knowledge, the present study is the first to uncover a potential relationship between serum LDL-C levels and lymphatic vessel remodeling in both non-xanthomatous and xanthomatous skin. The positive correlation between serum LDL-C levels and lymphatic branching, coupled with the identification of Ki-67-positive lymphatic endothelial cells in skin with elevated LDL-C, provides direct evidence for active lymphangiogenesis in hypercholesterolemic skin (Kyzas et al, 2005; Itai et al, 2024; Tan et al, 2024).

Prior studies demonstrated that severe hypercholesterolemia in mouse model causes lymphatic dysfunction and that oxidized LDL-C can directly suppress lymphatic endothelial cell proliferation (Lim et al, 2009; Singla et al, 2021). In contrast, our study demonstrated that lymphangiogenesis in hypercholesterolemic human skin is indirectly mediated by VEGF-C secreted from macrophages. Several lines of evidence indicate that macrophage-secreted VEGF-C is critically associated with lymphatic remodeling in inflammation, lymphedema and cancer metastasis (Ding et al, 2012; Gousopoulos et al, 2017; Itai et al, 2024). Interestingly, although VEGF-C production is often associated with alternatively activated M2 macrophages in tissue repair contexts (Lee et al, 2013), our findings demonstrate that increased M1 macrophage infiltration coincides with elevated VEGF-C expression, consistent with conditions including pterygium and renal fibrosis (Lee et al, 2024; Zhang et al, 2021). This suggests that in hypercholesterolemic inflammatory lesions, pro-inflammatory macrophages may contribute to VEGF-C mediated lymphangiogenesis, highlighting the context-dependent plasticity of macrophage-driven lymphatic remodeling. This is also consistent with the established mechanism in atherosclerosis, where VEGF-C is abundantly expressed in foamy macrophages (Nakano et al, 2005; Taher et al, 2016). Additionally, mouse studies examined severe systemic hypercholesterolemia with acute exposure, whereas our human subjects exhibit moderate, naturally occurring elevated LDL-C with chronic exposure. The increased lymphangiogenesis we observe may represent a long-term adaptive response attempting to maintain tissue homeostasis and facilitate lipid clearance.

The parallel findings in non-xanthomatous and xanthomatous hypercholesterolemic skin are particularly significant, as they suggest a common underlying pathogenic mechanism. The presence of similar lymphangiogenic features in both tissue types indicates that xanthomatous lesions may represent an extreme manifestation of the same biological processes occurring in non-xanthomatous hypercholesterolemic skin. These findings challenge traditional xanthoma formation models focused solely on foam cell accumulation. Enhanced lymphangiogenesis may initially compensate for impaired cholesterol clearance, but chronic stimulation could lead to lymphatic dysfunction, creating a pathological cycle where poor drainage exacerbates local cholesterol accumulation and inflammation.

In conclusion, our findings indicate that elevated serum LDL-C levels drive a complex cascade of pathological changes in human skin through three interconnected mechanisms: progressive lipid accumulation, macrophage-mediated inflammation, and aberrant lymphangiogenesis. The identification of active lymphangiogenesis is, to our knowledge, a previously unreported finding, implying that xanthomas may exist on a pathological continuum rather than as distinct entities. Thus, these mechanistic insights provide a clear shift in our understanding of how hypercholesterolemia affects peripheral tissues beyond the traditional cardiovascular manifestations.

### Limitations of the study

This study has several limitations that warrant consideration. The relatively small xanthomataous skin sample size (n = 7), though reflecting the rarity of these lesions, limits statistical power and generalizability. Moreover, our tissue samples were primarily allocated for histological analyses, precluding cytokine/chemokine profiling, which would provide functional validation of the inflammatory environment associated with M1 macrophage enrichment. The cross-sectional design precludes assessment of temporal relationships and causality between LDL-C elevation and tissue changes. We did not perform functional lymphatic assessments to determine whether observed lymphangiogenesis represents compensation or dysfunction. Future studies should include larger multi-center cohorts, longitudinal designs with treatment interventions, and functional lymphatic assessments to address these limitations and validate our findings.

## Materials and Methods

### Human skin samples

Upper eyelid skin samples were obtained from subjects undergoing elective blepharoplasty for ptosis. Xanthelasma palpebrarum lesions were acquired from patients (aged 62 ± 13 years) whose serum LDL was in the range of 117 ± 40 mg/dL. All samples were obtained at the Chiba University Hospital (Chiba, Japan) with the approval of the Institutional Review Board of the Chiba University Hospital and the Shiseido Global Innovation Center. All subjects provided written informed consent.

### Histology, immunofluorescence, and immunohistochemistry staining

Human skin samples were fixed with 4% paraformaldehyde and embedded in optimal cutting temperature compound. 10 μm cryosections were used for H&E staining using standard procedures and histological analysis. 10 μm or 50 μm thick sections were stained for immunohistochemical and immunofluorescence as previously described (Kajiya et al, 2007) using rabbit antibodies against Ki-67 (clone SP6, Abcam, ab16667), claudin-1 (Invitrogen, #71-7800), CD86 (clone EPR21962, Abcam, ab239075), VEGF-A (clone EP1176Y, Abcam, ab52917), VEGF-C (Santa-cruz, sc-9047), K14 (BioLegend, 905304) and LOX-1 (Abcam, ab60178), mouse monoclonal antibodies against claudin-4 (clone 3E2C1, Invitrogen, #32-9400), CD68 (clone KP1, Abcam, ab955), podoplanin (clone D2-40, Dako Cytomation, #M3619), K10 (clone DE-K10, Abcam, ab9026) and goat polyclonal antibodies against CD206 (R&D Systems, AF2535) and VEGF-C (Santa Cruz, sc-1881). Corresponding secondary antibodies were labeled with AlexaFluor 488, AlexaFluor 568 or AlexaFluor 647 (Invitrogen). Whole-mount immunostaining of lymphatic vessels was performed as previously described (Itai et al, 2024) using mouse mAb against podoplanin (clone D2-40, Dako Cytomation, #M3619). Tissues were cleared using Rapiclear 1.52 (SunJin Lab Co.).

### Image acquisition and quantitative analysis

The images of stained samples were captured with a confocal microscope (LSM880, Carl Zeiss). All quantitative analyses of skin samples were performed by a single investigator who was blinded to serum LDL-C values during the measurement process. Clinical information was revealed to the investigator after completion of image quantification to perform subsequent correlation analyses. Epidermal thickness was measured from H&E-stained tissue sections by drawing perpendicular lines from the basal layer to the top of the stratum granulosum at 10 randomly selected locations per section. For immunohistochemical and immunofluorescence quantification, up to three randomly selected high-power field images (100x, 200x, or 400x magnification) were analyzed per skin sample using ImageJ/FIJI software (National Institute of Health), avoiding areas with obvious tissue damage, folding artifacts, or edge effects. Epidermal parameters measured included: Ki67^+^ cells as a percentage of total epidermal cells, K14 and K10 mean fluorescence intensity in basal and suprabasal layers, respectively, expressed as K14/K10 ratio, and LOX-1 expression normalized to epidermal area. Dermal macrophage populations were quantified as: M1 (CD86^+^CD68^+^) and M2 (CD206^+^CD68^+^) macrophages per mm^2^ of dermis, expressed as M1/M2 ratio; and percentage of VEGF-A^+^ or VEGF-C^+^ cells among total CD68^+^ macrophages using automated image analysis. In whole-mount immunostaining of lymphatic vessels, the branching points of lymphatic capillaries in the upper dermal area within 300 μm depth from the epidermis were quantified using Imaris software (Bitplane).

### Statistical analysis

All statistical analyses were performed using GraphPad Prism 10.5.0 (GraphPad Software). For scatter plots, Student’s *t*-test for the Pearson correlation coefficient was applied. A *P*-value < .05 was considered statistically significant.

## Ethics Statement

Ethical approval for the study was obtained from the Institutional Review Board of the Chiba University Hospital and the MIRAI Technology Institute, Shiseido Co., Ltd. All human subjects provided full written, informed consent.

## Data Availability Statement

The data that support the findings of this study are available from the corresponding author (enkhtuul.gantumur@shiseido.com) upon request.

## ORCIDs

Enkhtuul Gantumur: http://orcid.org/0000-0002-1549-6374

Nao Itai: http://orcid.org/0000-0003-3552-2303

Kazuki Takagaki: http://orcid.org/0000-0002-8153-8077

Nobuyuki Mitsukawa: http://orcid.org/0000-0002-0234-8241

Shinsuke Akita: http://orcid.org/0000-0001-7075-6602

Kentaro Kajiya: http://orcid.org/0000-0002-5224-7051

## Conflict of Interest

EG, NI, KT, and KK are employees of Shiseido Co., Ltd. All other authors state no conflict of interest.
